# The role of host traits and geography in shaping the gut microbiome of insectivorous bats

**DOI:** 10.1128/msphere.00087-24

**Published:** 2024-03-21

**Authors:** Wentao Dai, Haixia Leng, Jun Li, Aoqiang Li, Zhongle Li, Yue Zhu, Xiaolin Li, Longru Jin, Keping Sun, Jiang Feng

**Affiliations:** 1 Jilin Provincial Key Laboratory of Animal Resource Conservation and Utilization, Northeast Normal University, Changchun, China; 2 Key Laboratory of Vegetation Ecology, Ministry of Education, Changchun, China; 3 Guangdong Provincial Key Laboratory of Silviculture, Protection and Utilization, Guangdong Academy of Forestry, Guangzhou, China; 4 School of Life Sciences, Central China Normal University, Wuhan, China; 5 College of Life Science, Jilin Agricultural University, Changchun, China; University of Wisconsin-Madison, Madison, Wisconsin, USA

**Keywords:** gut microbiome, diet composition, insectivorous bats, 16S rRNA, high-throughput sequencing

## Abstract

The gut microbiome is a symbiotic microbial community associated with the host and plays multiple important roles in host physiology, nutrition, and health. A number of factors have been shown to influence the gut microbiome, among which diet is considered to be one of the most important; however, the relationship between diet composition and gut microbiota in wild mammals is still not well recognized. Herein, we characterized the gut microbiota of bats and examined the effects of diet, host taxa, body size, gender, elevation, and latitude on the gut microbiota. The cytochrome C oxidase subunit I (COI) gene and 16S rRNA gene amplicons were sequenced from the feces of eight insectivorous bat species in southern China, including *Miniopterus fuliginosus*, *Aselliscus stoliczkanus*, *Myotis laniger*, *Rhinolophus episcopus*, *Rhinolophus osgoodi*, *Rhinolophus ferrumequinum*, *Rhinolophus affinis,* and *Rhinolophus pusillus*. The results showed that the composition of gut microbiome and diet exhibited significant differences among bat species. Diet composition and gut microbiota were significantly correlated at the order, family, genus, and operational taxonomic unit levels, while certain insects had a marked effect on the gut microbiome at specific taxonomic levels. In addition, elevation, latitude, body weight of bats, and host species had significant effects on the gut microbiome, but phylosymbiosis between host phylogeny and gut microbiome was lacking. These findings clarify the relationship between gut microbiome and diet and contribute to improving our understanding of host ecology and the evolution of the gut microbiome in wild mammals.

**IMPORTANCE:**

The gut microbiome is critical for the adaptation of wildlife to the dynamic environment. Bats are the second-largest group of mammals with short intestinal tract, yet their gut microbiome is still poorly studied. Herein, we explored the relationships between gut microbiome and food composition, host taxa, body size, gender, elevation, and latitude. We found a significant association between diet composition and gut microbiome in insectivorous bats, with certain insect species having major impacts on gut microbiome. Factors like species taxa, body weight, elevation, and latitude also affected the gut microbiome, but we failed to detect phylosymbiosis between the host phylogeny and the gut microbiome. Overall, our study presents novel insights into how multiple factors shape the bat’s gut microbiome together and provides a study case on host-microbe interactions in wildlife.

## INTRODUCTION

The guts of mammals harbor diverse and complex microbial communities that have profound impacts on host health and physiology (1, 2). Numerous studies have demonstrated that gut microbial communities play essential roles in driving nutrient acquisition (3), energy supply (4), behavioral regulation (5), and pathogen defense (6). Meanwhile, the composition and function of the gut microbiome are determined by several factors such as diet, phylogeny, geography, age, and the environment (7–9). Determining the relevant factors is essential for understanding how the mammalian gut microbiome adapts to complex and dynamic environments (1, 2).

Diet is one of the most important factors shaping gut microbiome in mammals by providing different nutrients to support the growth of specific microorganisms (9, 10). Wild animals living in natural conditions must capture enough food with nutrients to maximize their chances of survival, and over a long-term evolutionary process, their gut microbiome may have developed unique functions to adapt to the local food resources (4, 11). For example, cellulose-degradation in enzymes encoded by the gut microbiome of giant pandas (*Ailuropoda melanoleuca*) can facilitate the digestion and use of nutrients from bamboo (3), while gut microbiota of black howler monkeys (*Alouatta pigra*) provide additional energy and nutrients to compensate for seasonal fluctuations in diet (4). However, many studies have assessed the effects of influencing factors on gut microbiome only in one dimension, such as low or high fiber content (12) or the composition of different types of food (13). Most wild animals consume a variety of food types rather than focusing on a single type of food to meet nutritional demands. Furthermore, most current research on mammals has focused on humans (14), nonhuman primates (4), laboratory model animals such as mice (15), domestic animals including pigs, sheep, and cows (16–18), or captive animals (11) rather than wild animals. Conducting research on wild animals could provide valuable insights into the role of gut microbiome in natural conditions in terms of the evolution and adaptation of the host (1). However, observing foraging behavior is not a common way to identify the diet of mammals in the wild. Thus, there is a paucity of knowledge about the relationship between dietary composition and gut microbiome in wild mammals (1, 11).

In addition to diet, host species and their areas of distribution can also have important effects on gut microbiome. In some wild animals, a phylosymbiosis exists between the host phylogeny and its gut microbiome, which creates a congruence between the differences in bacterial communities and the phylogenetic divergences among species, that is, closely related species may feed on similar foods and have similar gut microbiota (8, 19). Characteristics of the host such as body size and gender may have a significant effect on the gut microbiome (8, 20). In addition, the different regions where the hosts are distributed may lead to differences in the gut microbiome due to different food and environmental factors (21). For example, elevational differences affect food composition and gut microbiome among populations of plateau pika (*Ochotona curzoniae*) (7), wild house mice (*Mus musculus domesticus*) (22), and black bears (*Ursus thibetanus*) (23). Therefore, various factors should be considered to reflect the adaptation of the gut microbiome of wild animals more comprehensively (2, 24).

Bats are the only mammals capable of flight, with more than 1,400 species worldwide (24, 25). About 70% of the species feed on insects, and the diet composition differs significantly among species (26). Bats provide important ecological services such as pest control in the fields of agriculture and forestry (25). Differences in body size, feeding strategies, feeding areas, and echolocation calls between different species facilitate their ability to achieve precise control over different types of insects during predation, even with a sympatric distribution (27, 28). Consequently, different food compositions may affect the composition or function of gut microbiome in insectivorous bats. Thus, insectivorous bats may serve as an ideal model for studying the evolution of host-gut microbiome relationships and the role of ecological factors influencing them. Nevertheless, studies on the relationship between gut microbiome and food composition in insectivorous bats are still limited. The host species is an important factor influencing the variation in gut microbiome for numerous species in different families (29, 30). However, the study of phylosymbiosis between host taxonomy and gut microbiome presents conflicting results concerning the role of bat phylogeny in shaping the microbiome, which may be explained by the different sample types, such as the gastrointestinal tract or feces (8, 29, 30). In addition, relationships exist between gut microbiome and changes in food resources, physiological periods, and geography (31, 32). However, most studies involving diets have roughly categorized diets, such as insectivores, frugivores, nectarivores, carnivores, sanguivores, and omnivores (8, 9). Furthermore, the absence of detailed food composition information for insectivorous bat species across various locations has resulted in an inability to understand the effects of ecological features, such as locality and elevation, on the gut microbiome (24).

The present study used 16S rRNA amplicon sequencing to investigate gut microbiome of eight species of insectivorous bats from four sites: *Miniopterus fuliginosus*, *Aselliscus stoliczkanus*, *Myotis laniger*, *Rhinolophus episcopus*, *Rhinolophus osgoodi*, *Rhinolophus ferrumequinum*, *Rhinolophus affinis*, and *Rhinolophus pusillus*. The cytochrome oxidase subunit I (COI) genes were sequenced to clarify the food composition of bats and to elucidate the factors driving the composition of the bat gut microbiomes, especially the effects of food composition. The objectives of this study were (i) to clarify the composition of the gut microbiome of insectivorous bats; (ii) to elucidate the effects of food composition on the gut microbiome of these bats, and (iii) to assess the effects of host and geography on the gut microbiome.

## RESULTS

### Composition of gut microbiome

**Fig 1:**
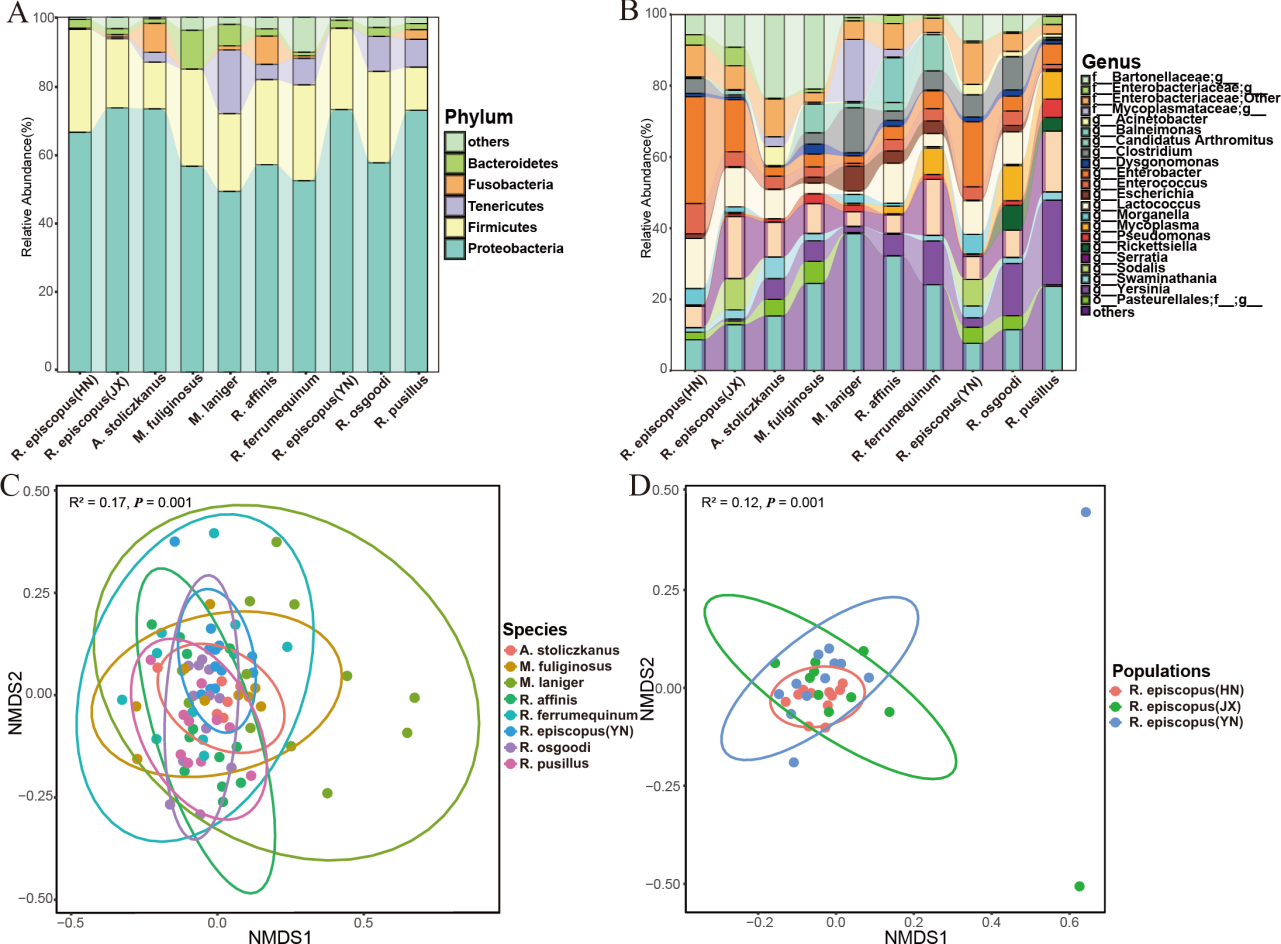
Plots showing the composition of the gut microbiome for each bat species at the (**A**) phylum and (**B**) genus levels. Nonmetric multidimensional scaling analysis of the beta diversity of the gut microbiomes among (**C**) samples per species, and (**D**) different geographic populations of *R. episcopus*. *R. episcopus* (HN), Hunan’s *Rhinolophus episcopus; R. episcopus* (JX), Jiangxi’s *Rhinolophus episcopus; R. episcopus* (YN), Yunnan’s *Rhinolophus episcopus*.

A total of 5.6 million quality sequences were generated (mean = 52,821 reads/sample, mean length = 422 bp), comprising 2,600 operational taxonomic units (OTUs). The dominant phyla (relative read abundance > 1%) of gut microbiota were Proteobacteria (64.42%), Firmicutes (22.28%), Tenericutes (5.10%), Bacteroidetes (3.20%), and Fusobacteria (2.10%) (Fig. 1A). The gut microbiome of bat species differed significantly in Fusobacteria (chi-squared = 22.11, *P* = 0.002) and Tenericutes (chi-squared = 24.72, *P* = 0.003) (Table S2). The dominant genera (relative read abundance > 1%) of gut microbiome were *Serratia* (9.80%), *Enterobacter* (8.99%), Bartonellaceae (7.33%), *Yersinia* (7.31%), and *Lactococcus* (7.12%) (Fig. 1B). The bat species differed significantly in the relative read abundances of these genera (Table S2). The gut microbiome did not differ significantly between *R. episcopus* populations at the phylum level but did differ significantly in the major genera (Table S2).

**Fig 2:**
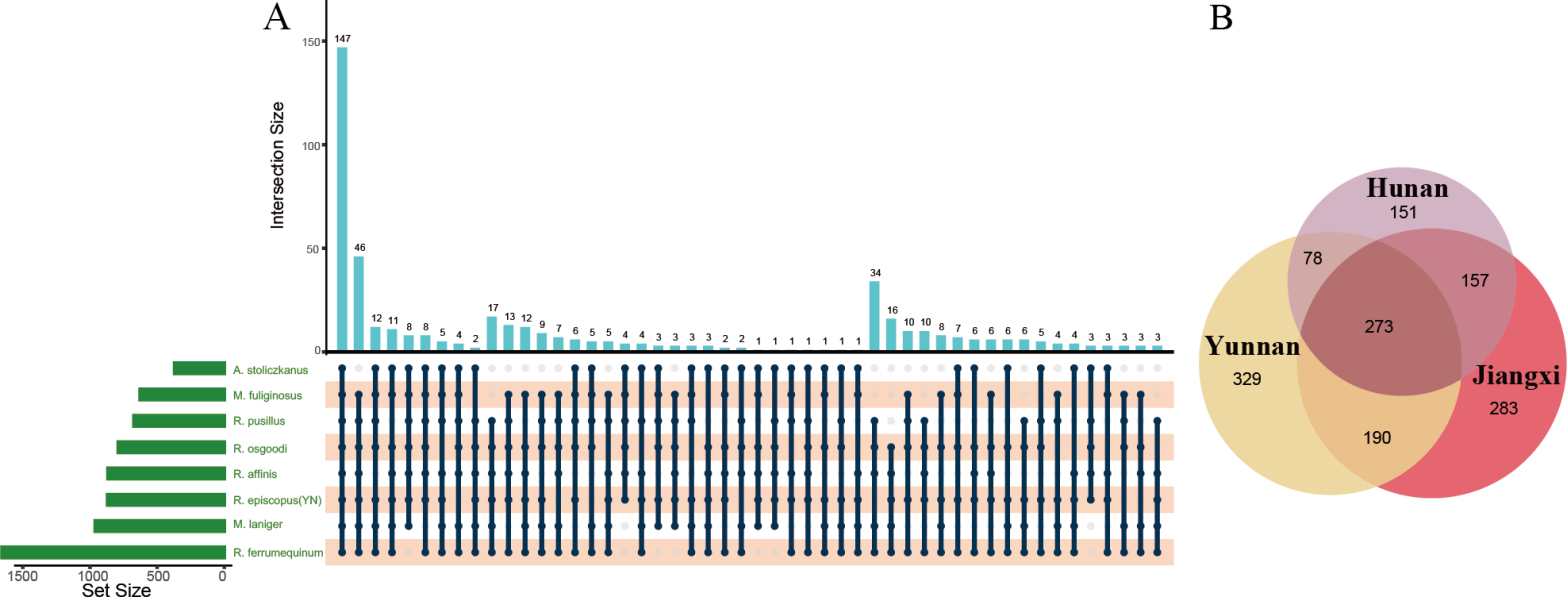
OTU overlap plots of gut microbiomes between bat species. (A) The UpSet plot of OTU overlap between all bat species; the upper bar indicates the number of overlapping OTUs, points crossed by a connecting line at the bottom indicate the overlapping species and the species are indicated at the left; (B) Venn diagram of OTU overlap of three *R. episcopus* populations.

UpSet analysis showed that 147 OTUs (5.65%) were shared by all bat species, and 273 OTUs (18.68%) were shared by three *R. episcopus* populations (Fig. 2A and B). Core microbial analysis showed that 15 OTUs were present in more than 80% of individuals, and all had relative abundances greater than 0.1% (Table 1). No significant differences were observed in alpha diversity of gut microbiome among bat species (Kruskal–Wallis test, Shannon diversity, chi-squared = 5.06, *P* = 0.65; observed OTUs, chi-squared = 4.86, *P* = 0.67, Fig. S1A and B). The Shannon diversity of gut microbiome showed no significant differences among *R. episcopus* populations [analysis of variance (ANOVA), F = 1.21, *P* = 0.31], but the observed OTUs were significantly different between Hunan and Jiangxi populations (Kruskal–Wallis test, chi-squared = 7.34, *P* = 0.02, Fig. S1C and D). Beta diversity analysis indicated significant differences in gut microbiomes among the bat species and among the *R. episcopus* populations [permutational multivariate analysis of variance (PERMANOVA), R^2^ = 0.17, *P* = 0.001; Fig. 1C; R^2^ = 0.12, *P* = 0.001; Fig. 1D; Table S3]. At the family level, there were significant differences between all family pairs (all *P* < 0.01) except for Hipposideridae (*A. stoliczkanus*) and Miniopteridae (*M. fuliginosus*) (Table S3; Fig. S2; R^2^ = 0.07, *P* = 0.401).

**TABLE 1:** Core gut microbiome present in 80% of bat individuals

OTU ID	Phylum	Family	Genus	Samples	Ratio	Frequency
OTU9	Proteobacteria	Enterobacteriaceae	*Enterobacter*	106	1	0.094
OTU12	Firmicutes	Enterococcaceae	*Enterococcus*	105	0.99	0.025
OTU62	Proteobacteria	Enterobacteriaceae	*–*	104	0.98	0.047
OTU3	Proteobacteria	Enterobacteriaceae	*Serratia*	102	0.96	0.070
OTU435	Firmicutes	Enterococcaceae	*Enterococcus*	102	0.96	0.007
OTU143	Proteobacteria	Enterobacteriaceae	*–*	101	0.95	0.006
OTU33	Proteobacteria	Moraxellaceae	*Acinetobacter*	98	0.92	0.007
OTU6	Proteobacteria	Enterobacteriaceae	*Yersinia*	97	0.91	0.071
OTU11	Proteobacteria	Acetobacteraceae	*Swaminathania*	94	0.88	0.018
OTU2	Firmicutes	Streptococcaceae	*Lactococcus*	92	0.86	0.046
OTU1	Proteobacteria	Bartonellaceae	*–*	91	0.85	0.062
OTU22	Proteobacteria	Enterobacteriaceae	*Serratia*	91	0.85	0.017
OTU36	Proteobacteria	Pseudomonadaceae	*Pseudomonas*	91	0.85	0.005
OTU4	Firmicutes	Streptococcaceae	*Lactococcus*	87	0.82	0.026
OTU619	Proteobacteria	Enterobacteriaceae	*Serratia*	86	0.81	0.004

### Diet composition of eight bat species

A total of 8.1 million quality sequences were generated (mean = 76,089 reads/sample), comprising 1,532 OTUs. There were no significant differences in alpha diversity of diet compositions among bat species (Kruskal–Wallis test, Shannon diversity, chi-squared = 8.89, *P* = 0.26; observed OTUs, chi-squared = 10.16, *P* = 0.17, Fig. S3A and B) or among *R. episcopus* populations (ANOVA, Shannon diversity, F = 1.21, *P* = 0.31; Kruskal–Wallis test, observed OTUs, chi-squared = 1.58, *P* = 0.45, Fig. S3C and D).

**Fig 3:**
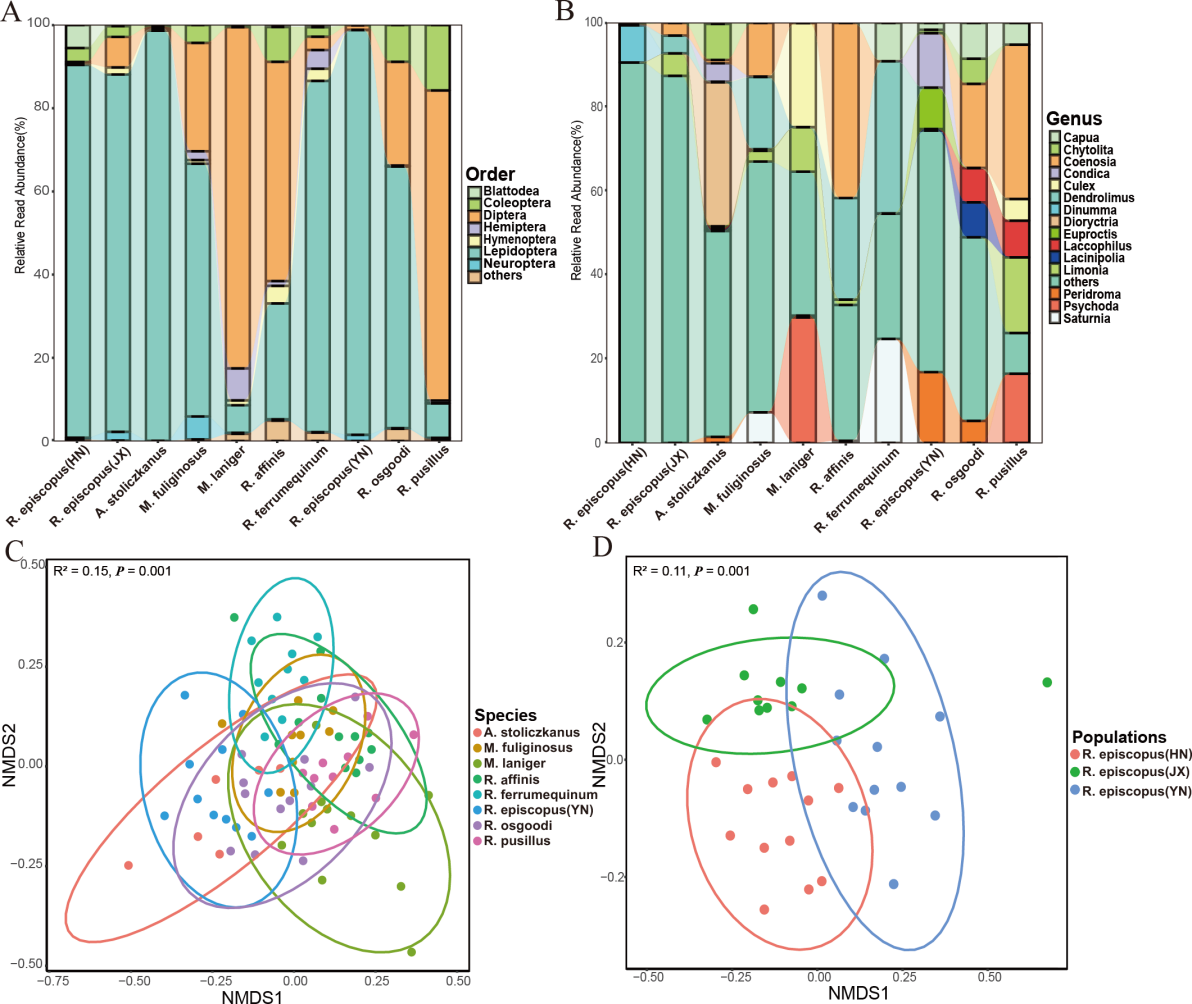
Plots showing the composition of the diet for each bat species at the (**A**) phylum and (**B**) genus levels. Nonmetric multidimensional scaling analysis of the beta diversity of the composition of diet among (**C**) samples per species, and (**D**) different geographic populations of *R. episcopus*.

Diets were composed completely of arthropods at the phylum level, while 93.99% were insects at the order level. A total of seven orders of insects were obtained, while significant differences were detected in the consumption of Coleoptera, Diptera, Hemiptera, and Lepidoptera insects by each bat species (Fig. 3A; Table S4, Kruskal–Wallis test, all *P* < 0.05). At the family level, a total of 20 major families were obtained, several of which differed significantly between species (Table S4, all *P* < 0.05). At the genus level, a total of 15 major genera were obtained, several of which differed significantly between species (Fig. 3B; Table S4, Kruskal–Wallis test, all *P* < 0.05). Beta diversity analysis showed significant differences in the composition of diets among bat species (PERMANOVA, R^2^ = 0.15, *P* = 0.001; Fig. 3C) and significant differences in diet composition among *R. episcopus* populations (PERMANOVA, R^2^ = 0.11, *P* = 0.001; Fig. 3D).

### Relationships among gut microbiome and predictor factors

**Fig 4:**
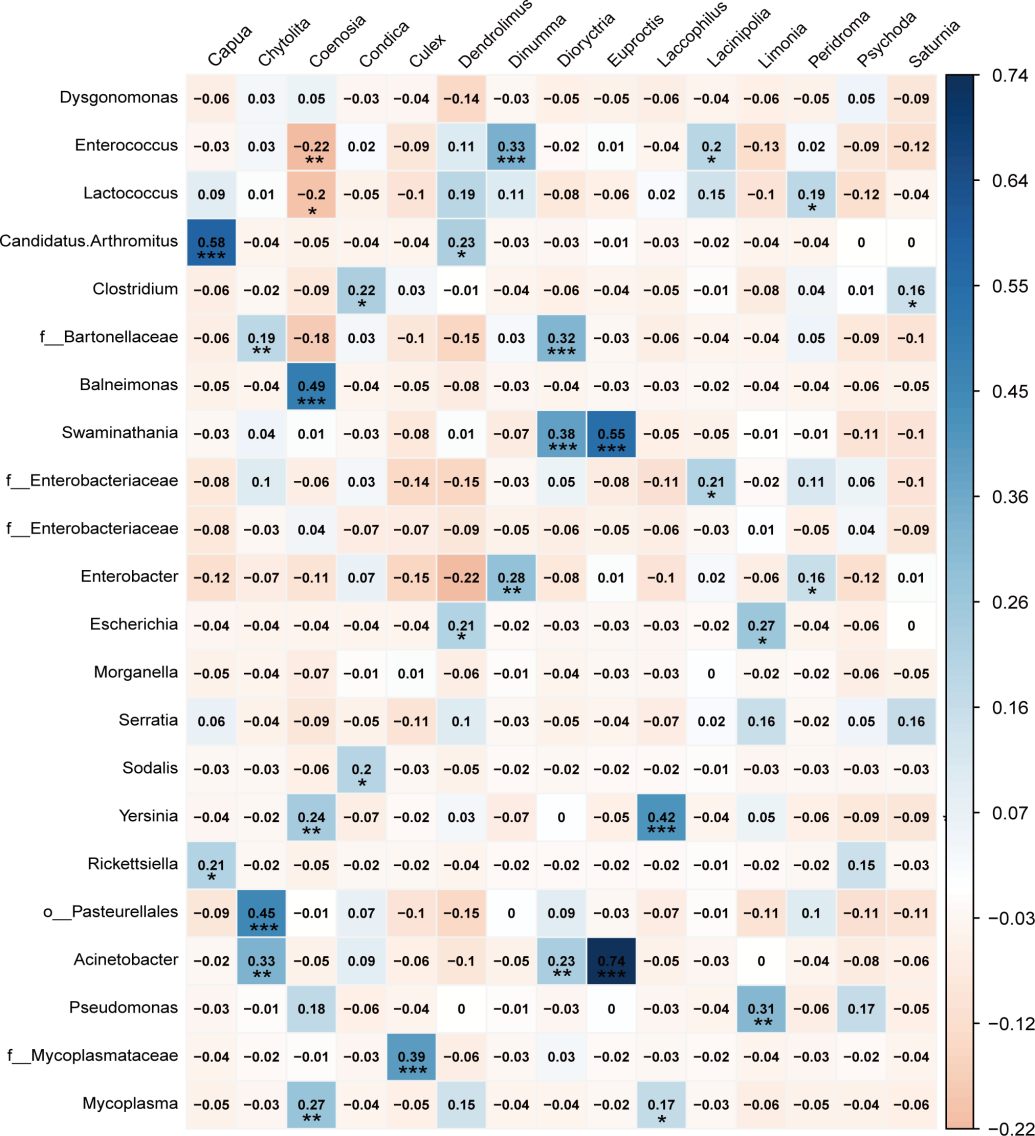
Correlation of the gut microbiome data set with the dietary data set at the genus level.

**Fig 5:**
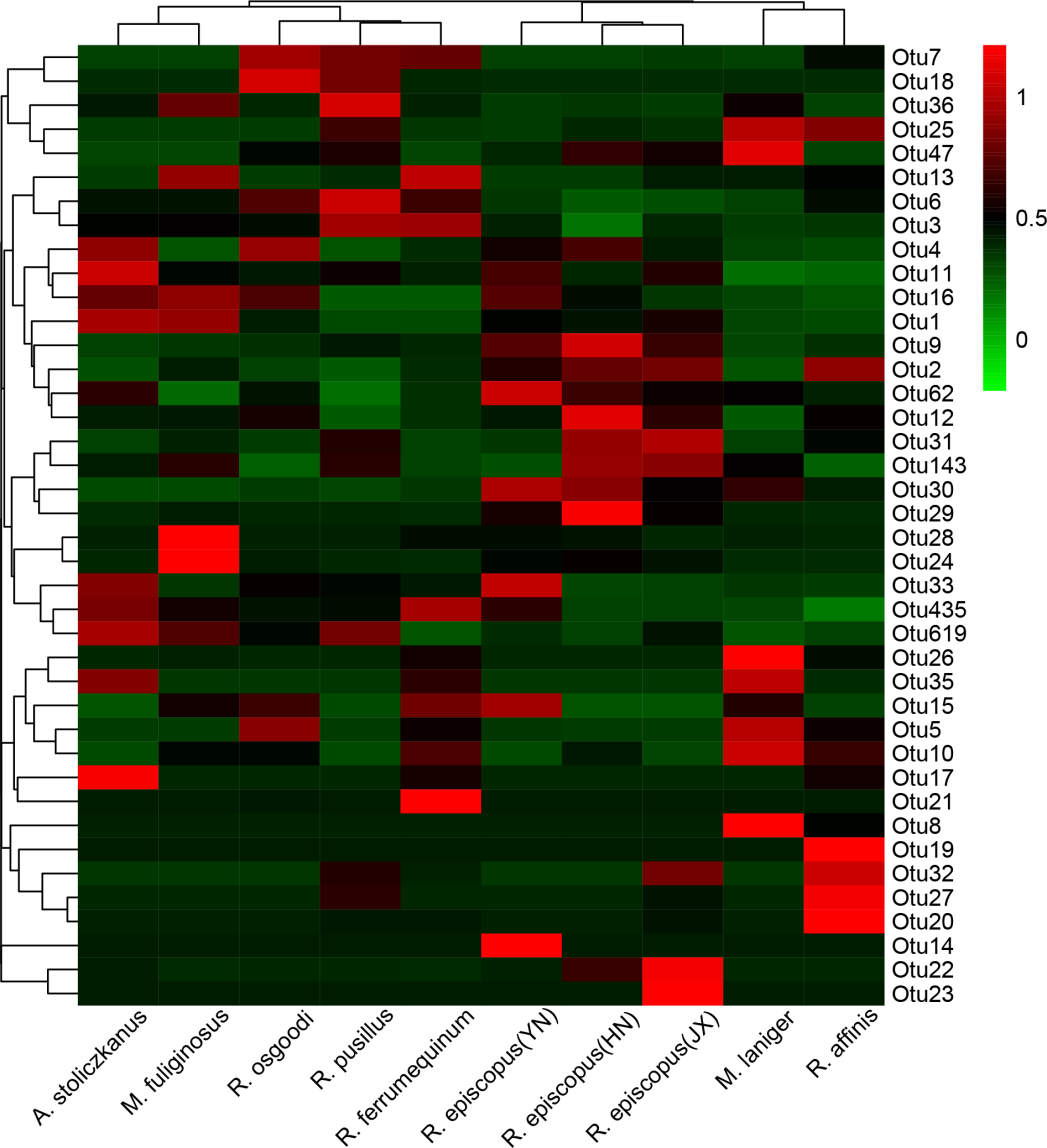
The heatmap of the 40 OTUs with the highest abundance in the gut microbiomes of eight insectivorous bats.

The results of the generalized linear model (GLM) showed that elevation, latitude, body weight of bats, diet composition of different taxonomic levels, and host taxa were significant predictors of the gut microbiome (Fig. 4; Table S5). The Mantel test showed a significant association between bat family and gut microbiome (r = 0.11, *P* = 0.02). PERMANOVA analyses revealed that the host species (R^2^ = 0.19, *P* = 0.0001) is a significant factor explaining microbial variation among all bat species, but not for gender (R^2^ = 0.012, *P* = 0.06), while the interaction of host and gender had a significant effect on the gut microbiome (R^2^ = 0.07, *P* = 0.009). Furthermore, the host species (R^2^ = 0.12, *P* = 0.0001) was a significant factor explaining diet variation among all bat species, but not for gender (R^2^ = 0.006, *P* = 0.95) or the interaction of host and gender (R^2^ = 0.04, *P* = 1.00). The results of the Mantel test showed that bat species with geographic overlap or similar diets had significantly similar gut microbiomes (Table S6, all *P* = 0.01). The results of Procrustes analysis revealed significant correlations between gut microbiome and diet at different taxonomic levels (Table 2, *P* < 0.05). Mantel tests between the host phylogenetic distance and the weighted, as well as unweighted, UniFrac gut microbiome dissimilarity values revealed no significant correlation between the gut microbiome and host phylogeny (weighted UniFrac, *r* = 0.08, *P* = 0.39; unweighted UniFrac, *r* = 0.15, *P* = 0.67). The heatmap of those bacterial OTUs with relatively high abundances showed the *Rhinolophus* bat species did not cluster together (Fig. 5). These results implied no convergence between the gut microbiome and the evolutionary relationship with the host.

**TABLE 2:** Procrustean correlations of the gut microbiome and dietary, both summarized at different taxonomic levels

	OTU	Genus	Family	Order
M^2^	0.965	0.956	0.929	0.927
*P* value	0.001	0.017	0.007	0.003

## DISCUSSION

### Composition of gut microbiome

Consistent with previous findings in insectivorous bats (9, 32, 33), this study found gut microbiome at the phylum level consisted mainly of Proteobacteria, Firmicutes, Tenericutes, Bacteroidetes, and Fusobacteria. Proteobacteria (relative abundance > 50%) was the most abundant bacterial phylum, which is similar to the previous studies on Phyllostomid, Emballonuridae, and Pteropodidae bats (over 40% relative abundance) (9, 34), but has low relative abundance (usually less than 5%) in terrestrial mammals such as mice (35) and humans (36). Firmicutes is a specific bacterial phylum distributed in all mammals that plays an important role in the digestion and catabolism of food, especially of polysaccharides, which can promote calorie extraction from food and produce large amounts of short-chain fatty acids to influence nutrient acquisition and energy regulation (15, 37). The Bacteroidetes are strictly anaerobic bacteria, similar to findings in birds (38, 39). This phenomenon reflects the co-evolution of gut microbiome and host physiology, as gut microbiomes of bats and birds have many special features related to the gastrointestinal system and flight activity not found in many other animals (9, 40). Bacteroidetes are also related to the high level of carbohydrates and fermentation of sugar molecules to provide nutrients (35, 36). Tenericutes constituted a small portion of the microbiome in this study, possibly because the samples were collected in the summer rather than the fall, a period when members of this phylum are most active during periods of rapid fat accumulation (9, 38). Fusobacteria is often associated with intestinal-like diseases, being one of the indicator species of health status. These bacteria produce butyrate with known positive effects on the control of enteric pathogens (6, 41).

At the genus level, *Enterobacter* (Enterobacteriaceae), *Enterococcus* (Enterococcaceae), and *Lactococcus* (Streptococcaceae) accounted for a large proportion of the gut microbiome. These groups are short-chain fatty acids (SCFA) fermenters involved in the microbial fermentation of carbohydrates and produce large quantities of SCFAs to maximize energy (42). This suggests that bat’s gut microbiome tends to consist of facultative anaerobes rather than strictly obligate anaerobes. Chitinase-producing bacteria were found in this study, including *Serratia marcescens* (Firmicutes), *Bacillus cereus* (Firmicutes), and *Enterobacter cloacae* (Proteobacteria), all of which contribute to chitin degradation (43). The present study also identified several potential pathogenic bacteria such as *Yersinia*, *Pseudomonas*, *Bartonella*, *Serratia*, *Enterobacter*, *Clostridium*, and *Acinetobacter*, suggesting that bats may be a source of microorganisms that are potentially pathogenic to humans and animals (44–48).

### Factors influencing gut microbiome of insectivorous bats

Clarifying diet composition can help researchers understand whether a link exists between food and gut microbiome. For example, changes in nutrient composition can affect the composition and function of the gut microbiome (4, 49), and feeding livestock animals with insects that are high in protein and lipid content can help promote growth performance (50). In this study, Fusobacteria had a significantly higher relative abundance in *R. affinis* than in other species, which may be related to it having large amounts of Dipteran insects in its diet, including insects such as the genus *Coenosia*, which carry large numbers of viruses (51). Procrustes analysis and GLM revealed strong correlations between diet composition and gut microbiome, indicating that dietary variations among eight bat species had a direct impact on gut microbiome. A particular diet composition can significantly affect the gut microbiome, possibly due to these microbiomes being enriched by a variety of foods to which the intestinal microbiota have been exposed for a long time (32), some microorganisms have gained a competitive advantage and become dominant during co-evolutionary processes (40, 52). Our findings provide a more accurate picture of the bat’s diet, implying the importance of regulating food composition for animal conservation.

Bat taxon was a significant factor in explaining the variation in the beta diversity of bacterial OTU levels, suggesting that the host taxon is an important driver of its gut microbiome, as in previous studies of many species at the family level (9, 30). Indeed, the gut microbiome of bats has been closely related to their hosts during evolution, and phylosymbiosis may exist in Phyllostomid, Emballonuridae, Molossidae, Mormoopidae, Noctilionidae, Natalidae, and Vespertilionidae bats (9, 30). However, no significant correlation was detected between the gut microbiome and the host phylogeny, implying the absence of phylosymbiosis. That means host identity, but not evolutionary history drove the changes in gut microbiota in this study. It has been shown that gut microbiomes carry phylogenetic signals from the host taxon of bats (53), but studies on different bat species yielded mixed results (8, 29, 30, 39). A possible reason is that intestinal mucosa retains more of the host’s evolutionary traits, whereas feces may be more reflective of the host’s ecological traits (30). Nevertheless, although Lutz et al. collected a variety of materials from bats (feces, gut lumen, skin, and oral samples), they found no correlation between host phylogeny and bacterial community dissimilarity (29). Another recent study also found little evidence of phylosymbiosis and bat hosts especially, bird-like gut microbiomes, potentially associated with the physiological adaptations to flight (39). This implies that host ecological traits, rather than host phylogeny, may drive the variation in microbial community. Bats and birds both contain relatively low proportions of Bacteroidetes but high proportions of Proteobacteria. Proteobacteria have high functional variability, which maximizes microbial function while reducing diversity and mass for more efficient flight (54). The shorter guts and retention time of bats could facilitate microbial exchange through an aerobic environment (55). In addition, high digestive efficiency and greater rates of intestinal paracellular absorption in bats compared to nonflying mammals could also aid in nutrient absorption (55, 56). The high energy demands of flight may have imposed a limitation on the bat gut microbiome, leading to the observed lack of correlation with the host phylogeny (29).

The weak effect of gender on gut microbiota may be caused by the small sample size and the avoidance of the bat’s reproductive period (8, 57). Although the bats in this study were all small-bodied mammals (<20 g), the body weight varied significantly among species (20, 53). The results showed that geography (elevation and latitude) was a significant factor influencing the variation of the gut microbiome, possibly due to the different food resources or climatic conditions of the sampling region. The Yunnan–Guizhou Plateau, where the Xianren and Longxu caves are located, is in the second order in the terrain of the Chinese mainland, having a relatively high elevation, whereas the Jiumen and Luohan caves are in the third order, with a lower elevation (58). The local ecosystem components among different collection localities may have influenced differences in the composition or function of the gut microbiome, allowing hosts to better adapt to local foods and environmental conditions (21). For example, differences in diet composition of two populations of lesser long-nosed bats (*Leptonycteris yerbabuenae*) distributed in different regions of Mexico determined the composition of microbial communities (31). The diet composition and gut microbiome of plateau pikas from the Tibetan Plateau varied with elevation, and a strong correlation between diet composition and gut microbiome was found (7). Environmental conditions at high elevations are characterized by cold, aridity, and low oxygen content, all factors that can affect the cardiovascular system and energy metabolism. Mammals respond to environmental stresses, and gut microbiome adapts to high-altitude environments; for example, yak and Tibetan sheep rumen microbial communities at high elevations show convergence, with significantly lower production of methane and volatile fatty acids (59). Bats have a wide distribution and consume large amounts of energy during flight activity (60); an exploration of how gut microbiome adapts to flight at high elevations is necessary in the future. However, 16S rRNA gene amplicon sequencing used in this study also has limitations. First, the primers used for amplification could introduce a bias, as they bind to regions that are not 100% conserved across all bacteria (61, 62). Second, this approach is not possible to obtain the full-length sequence of the 16S rRNA gene (63). This could lead to the sequences only being identified to the genus level due to high similarity between 16S rRNA genes from closely related species. While the Illumina MiSeq sequencing platform has been demonstrated to produce reads with higher quantity and quality than other platforms (62, 63). Especially, we performed the appropriate quality controls to reduce the spurious and rare OTUs to minimize the influence of sequencing errors for this study.

In conclusion, we surveyed the gut microbiomes of eight insectivorous bats distributed in southern China and analyzed the effects of host traits, host taxonomy, geography, and phylogeny on shaping gut microbiomes. The results showed that gut microbiome was significantly correlated with diet at different taxonomic levels and also shaped predominantly by host traits and geography. In addition, no phylosymbiosis was observed between gut microbiome and host phylogeny. This study provides an opportunity for researchers to understand how these factors determine the nature of gut microbiome, with important implications for the conservation and management of wild animals.

## MATERIALS AND METHODS

### Sample collection

**Fig 6:**
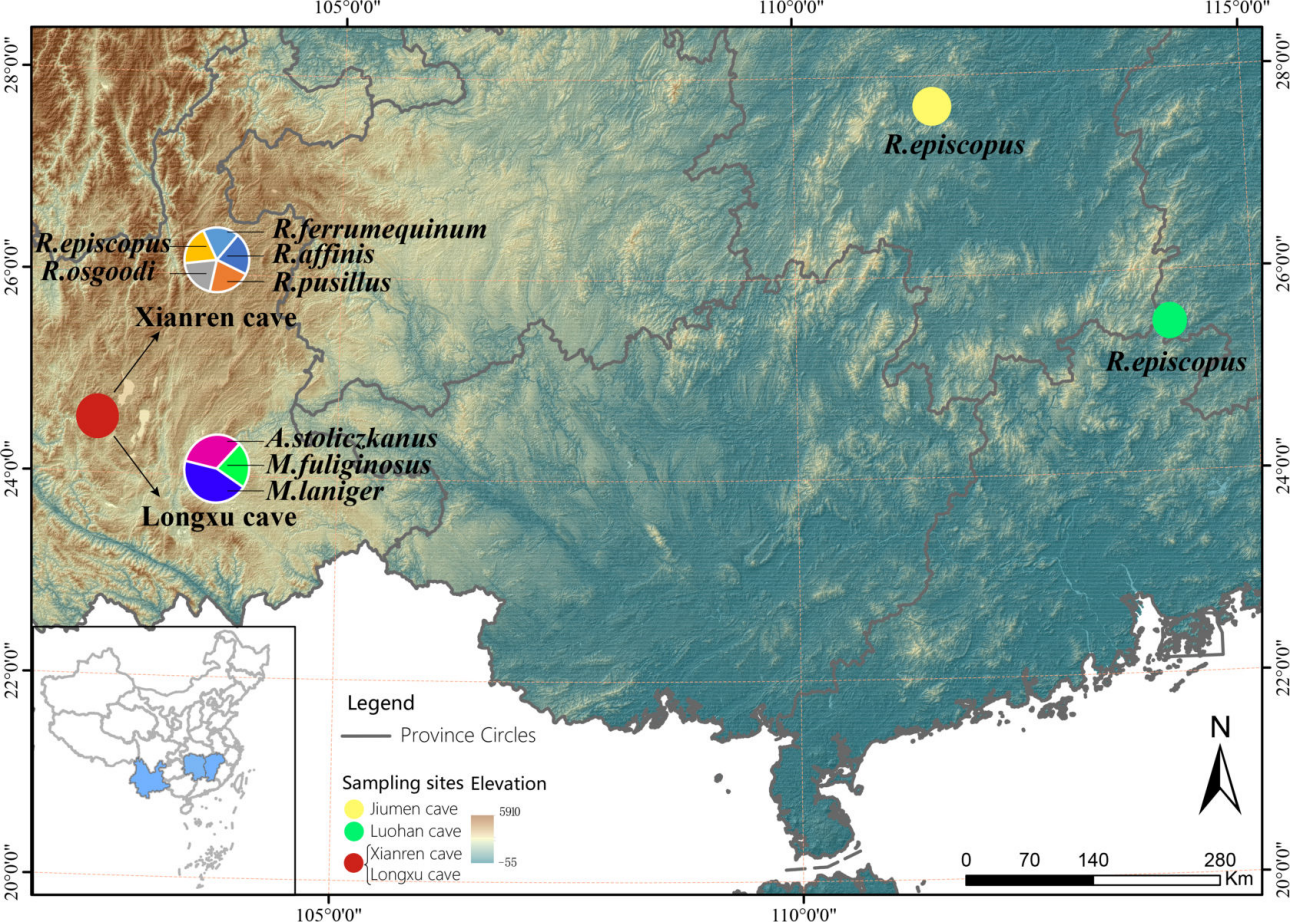
Map of sampling site distribution and elevation, with pie charts showing samples per species composition and proportions within habitats.

In July–August 2018, fecal samples were collected from four locations in southern China (Fig. 6). The Jiumen cave with about 200 m elevation is located in Lengshuijiang City, Hunan Province, and is surrounded by evergreen broad-leaved forest and rice farmland (64). The Luohan cave with about 450 m elevation is located in Ganzhou City, Jiangxi Province, and is surrounded by evergreen broad-leaved forest (65). The Longxu cave and Xianren cave with about 2,100 m elevation are located in Kunming City, Yunnan Province. These two caves are located on the Yunnan–Guizhou Plateau and are surrounded by semi-humid evergreen broad-leaved forest and farmland (tobacco and corn) (66). Eight bat species were collected, including *R. episcopus* from the Jiumen (*n* = 12) and Luohan caves (*n* = 10); *M. fuliginosus* (*n* = 9), *A. stoliczkanus* (*n* = 6), and *M. laniger* (*n* = 12) from the Longxu cave; *R. osgoodi* (*n* = 12), *R. ferrumequinum* (*n* = 11), *R. affinis* (*n* = 12), *R. pusillus* (*n* = 10), and *R. episcopus* (*n* = 12) from the Xianren cave. Given that the Longxu and Xianren caves are located just slightly over 1 km apart, the sympatric distribution of the bats in the two caves was documented for this study (Table 3). The collection was conducted during the nonmating and nonlactating periods. From 21:00 in the evening to 4:00 the following morning, bats were captured with mist nets at the entrance of each cave as they returned to the cave. Species and gender were identified based on external morphological characteristics, and bats were judged as juveniles or adults based on epiphyseal fusions (67). First, juveniles were immediately released *in situ* after being identified. Next, captured bats were placed individually in sterilized kraft paper bags for 2 h while droppings were collected. The bags were checked frequently to ensure the collection of fresh droppings. The droppings were placed into lyophilized tubes containing RNAlater (RNA-EZ Reagents RNA-Be-Locked A, Sangon Biotech, China) using sterilized forceps. At least five droppings per bat were collected and stored at −80°C for DNA extraction. The body mass was determined with an electronic balance (ProScale LC-50, Accurate Technology, Asheville, USA, 0.01 g). The elevation and latitude of the sampling sites were measured using a global positioning instrument (GPSmap60CSx, GARMIN, Shanghai, China) (Table 3). The bats were released at the cave entrance immediately after droppings collection and measurements were finished.

**TABLE 3:** Collection sites and species information of bats^*a*^

Site (Province)	Species	Family	Date	Number (n)	Elevation (m)	Latitude (N)	Weight (g)
Jiumen cave (Hunan)	*R. episcopus*	Rhinolophidae	12 July 2018	12 (F = 5, M = 7)	210	27°44′N	9.01 ± 0.93
Luohan cave (Jiangxi)	*R. episcopus*	Rhinolophidae	20 July 2018	10 (F = 4, M = 6)	442	25°27′N	9.79 ± 0.94
Xianren cave (Yunnan)	*R. episcopus*	Rhinolophidae	14 August 2018	12 (F = 4, M = 8)	2,084	24°30′N	6.42 ± 0.29
Xianren cave (Yunnan)	*R. osgoodi*	Rhinolophidae	14 August 2018	12 (F = 6, M = 6)	2,084	24°30′N	6.05 ± 0.51
Xianren cave (Yunnan)	*R. ferrumequinum*	Rhinolophidae	14 August 2018	11 (F = 6, M = 5)	2,084	24°30’ N	18.82 ± 1.25
Xianren cave (Yunnan)	*R. affinis*	Rhinolophidae	14 August 2018	12 (F = 4, M = 8)	2,084	24°30′N	13.29 ± 1.02
Xianren cave (Yunnan)	*R. pusillus*	Rhinolophidae	14 August 2018	10 (F = 2, M = 8)	2,084	24°30′N	4.90 ± 0.36
Longxu cave (Yunnan)	*M. fuliginosus*	Miniopteridae	16 August 2018	9 (F = 5, M = 4)	2,084	24°30′N	12.98 ± 0.89
Longxu cave (Yunnan)	*A. stoliczkanus*	Hipposideridae	16 August 2018	6 (F = 2, M = 4)	2,084	24°30′N	7.04 ± 0.58
Longxu cave (Yunnan)	*M. laniger*	Vespertilionidae	16 August 2018	12 (F = 3, M = 9)	2,084	24°30′N	5.25 ± 0.37

^*a*^ Note: Body weight is mean ± SD; F, females; M, males.

### DNA extraction, PCR amplification, and sequencing

Genomic DNA was extracted from guano using an E.Z.N.A. Mag-Bind Soil DNA Kit (OMEGA Bio-Tek, Norcross, USA) according to the manufacturer’s instructions. After DNA extraction, DNA integrity was tested using 2% agarose gel. Genomic DNA was accurately quantified using a Qubit 3.0 DNA Assay Kit (Life Technologies, Waltham, USA) to determine the amount of DNA in each sample. For the gut microbiome, the primers 341F (5′-CCTACGGGNGGCWGCAG-3′) and 805R (5′-GACTACHVGGGTATCTAATCC-3′) were used to amplify the V3–V4 region of the 16S rRNA gene. For dietary identification, we used the primers LCO-1490 (5′-GGTCAACAAATCATAAAGATATTGG-3′) and ZBJ-ArtR2c (5′-WACTAATCAATTWCCAAATCCTCC-3′) to amplify a 225 bp fragment of the COI gene (68, 69). The PCR systems and conditions have been described in previous studies (26, 70). The PCR products were purified using Agencourt AMPure XP beads (Beckman Coulter, CA, USA), and the DNA concentration of each sample was quantified using the Qubit 3.0 DNA assay kit (Life Technologies) to normalize the samples according to the manufacturer’s protocol. The final sequencing concentration was 20 pmol, and 10 ng of DNA was taken from each sample. The final products were sequenced using an Illumina Miseq platform (2 × 300 bp) at Sangon Biotech (Shanghai, China).

### 16S rRNA and COI sequence analysis

Primers and adaptors were removed from the raw sequences using cutadapt v1.2.1 (71), and paired-end reads were merged using PEAR v0.9.6 (72) based on barcode tags to distinguish samples. Finally, the data files were quality-filtered using Prinseq v0.20.4 (73). High-quality reads were obtained using the following criteria: no presence of ambiguous bases (N), no barcode sequence errors, and a minimum of five consecutive high-quality base pairs (Q ≥ 20), and a maximum of three consecutive low-quality base pairs were allowed. The OTUs were clustered using Usearch after the singletons and chimeras were removed (74). All optimized sequences were mapped to representative sequences, and OTU tables with a 97% similarity threshold were generated. For 16S rRNA data, species taxonomic information for each OTU was obtained using the Qiime1 v1.9.1 software and the Greengenes database. The OTU table was filtered using a minimum cluster size of 0.001% of the total reads to improve accuracy. Trees were constructed based on FastTree, samples with fewer than 23,600 sequences were discarded, and the data were rarefied to 23,600 sequences per sample. Finally, the alpha diversity (Shannon diversity and observed OTUs) of the gut microbiome of individuals was calculated, and the OTUs were classified according to species taxonomic level (75). For COI data, the rhinolophid bats collected from the Xianren cave were used in a previous study (26), in order to minimize the effects of an uneven number of sample sequences, the number of sequences per sample was diluted to 11,000. Finally, to minimize the effect of sequencing errors, the OTUs representing <0.1% of the normalized sequences for each sample were removed to prevent the generation of potentially erroneous results. Representative sequences of each OTU were manually compared with the reference sequences in the Barcode of Life Database (www.boldsystems.org/) in the “Species Level Barcode Records” database of ANIMAL IDENTIFICATION [COI] tool and the Genbank database (http://www.ncbi.nlm.nih.gov/GenBank) using BLASTN’s Nucleotide BLAST tool to obtain taxonomic information. The identification criteria were based on slightly modified “strict” and “best” matching methods (76, 77). The guidelines were (i) a solid match (>98.5%) to one species resulting in a species-level assignment. If there is a match to multiple species, all belonging to the same genus, this leads to a genus-level assignment. (ii) If there is a match (>98.5%) to more than one species from different genera, and only one of them belongs to China, it is considered a species-level assignment. (iii) A match (>98.5%) to several species from different genera within the same family or to reference sequences that are only identified to the family level, indicates a family-level assignment. Any OTUs that did not match any taxonomic information were excluded.

### Statistical analysis

We used ANOVA or Kruskal–Wallis tests to compare alpha diversity (Shannon diversity and observed OTUs) of gut microbiome among bat species. The Bonferroni correction was applied to the *P* values by post hoc Dunn’s multiple comparisons test in the FSA package. PERMANOVA was used to test the effects on host species and gender and their interaction effects on Bray–Curtis distance between the gut microbiome and diet using the “adonis” function in the vegan package. Composition and relative read abundance of the gut microbiome of each bat species at the phylum and genus levels were calculated. In addition, ANOVA or Kruskal–Wallis test with a post hoc Dunn’s test was performed on the major constituents to detect significant differences between species. Venn diagrams were used to represent the overlap in the distribution of OTUs among bat species and OTUs that were found in at least 80% of the samples were counted and defined as core OTUs using Usearch. Beta diversity between species was calculated with Bray–Curtis distance matrices and plotted using nonmetric multidimensional scaling (NMDS) in the phyloseq package at the OTU level and family level, and PERMANOVA was used to detect differences among bat species. The GLM with Poisson distribution was used to examine the effects of predictor variables on gut microbiome. Elevation, latitude, the body weight of bats, diet composition of different taxonomic levels, and host taxa (family and genus) were assigned as the predictor variables. The variance inflation factors were calculated to identify the collinearity between the predictor variables until the values of all factors were less than 10. We chose the best-fitting GLM according to Akaike’s information criterion corrected for small sample size using the “dredge” function in the MuMIn package. We also performed a Mantel test to examine whether gut microbiome at the family level contained phylogenetic signals. The OTU table was transformed to present-absence data using the “decostand” function in the vegan package, and the bat family was transformed using Gower distance in the cluster package. The significance of r values was assessed via 999 permutations.

For the analysis of dietary data, alpha diversity was tested as described above. Diet composition and relative read abundance for each bat species were also calculated and tested as described above at the order, family, and genus levels. Beta diversity between species was calculated with Bray–Curtis distance matrices using NMDS with PERMANOVA to detect differences among bat species. To assess the effects of geographic variation on gut microbiome, the diet composition and gut microbiome of three *R. episcopus* populations were analyzed using the method described above.

Based on the results for beta diversity of species that had similar diets or geographic overlap, we used the Mantel test to demonstrate correlations of gut microbiome between species at the species level. Procrustes analysis was used to test for consistency in the composition of prey and gut microbiota across taxonomic levels, using the “protest” function; a 999 permutation test was used to determine significance. To assess the influence of host phylogeny on the variability of gut microbiome, phylosymbiosis analysis was performed to evaluate the phylogenetic congruence between gut microbiomes and the hosts. The cyt *b* gene sequences of each bat species were downloaded from the NCBI database (Table S1) and aligned in Geneious prime (v.2022); four sequences were selected for each species, and there were variant loci between the sequences. Then, these sequences were grouped according to the species in MEGA 11, and finally, the Kimura 2-parameter model was selected to calculate the average phylogenetic distance between species. For gut microbiomes, weighted and unweighted UniFrac distances were used to obtain the dissimilarity distance matrix between individuals. After grouping according to species, the average dissimilarity distance matrix between species was calculated, after which a Mantel’s test with 999 permutations was performed. The membership of gut microbiome for the top 40 OTUs was revealed by a heatmap plot in the pheatmap package. All the data analyses were performed using R (v.4.1.1) unless otherwise stated (78), with the resulting data expressed as mean ± SD.

## Data Availability

The raw sequence data are submitted to the National Center for Biotechnology Information (NCBI) Sequence Read Archive under accession number PRJNA1025871.

## SUPPLEMENTAL MATERIAL

FIG. S1 msphere.00087-24-s0001.tif Alpha diversity of gut microbiome among bat species.

FIG. S2 msphere.00087-24-s0002.tif NMDS results.

FIG. S3 msphere.00087-24-s0003.tif Alpha diversity of diet composition among bat species.

Supplemental material legends msphere.00087-24-s0004.docx Legends for all supplemental items.

Table S1 msphere.00087-24-s0005.docx GenBank accession numbers.

Table S2 msphere.00087-24-s0006.xlsx Results of Kruskal-Wallis test and ANOVA.

Table S3 msphere.00087-24-s0007.xlsx Pairwise PERMANOVA results.

Table S4 msphere.00087-24-s0008.xlsx Results of Kruskal-Wallis tests and ANOVA.

Table S5 msphere.00087-24-s0009.docx GLM result.

Table S6 msphere.00087-24-s0010.docx Mantel test.

ASM does not own the copyrights to Supplemental Material that may be linked to, or accessed through, an article. The authors have granted ASM a non-exclusive, world-wide license to publish the Supplemental Material files. Please contact the corresponding author directly for reuse.
