# Supplementary figures and images for: The role of host traits and geography in shaping the gut microbiome of insectivorous bats
Source: mSphere. 2024 Mar 21;9(4):e00087-24. doi: 10.1128/msphere.00087-24 (PMC11036801; doi:10.1128/msphere.00087-24)

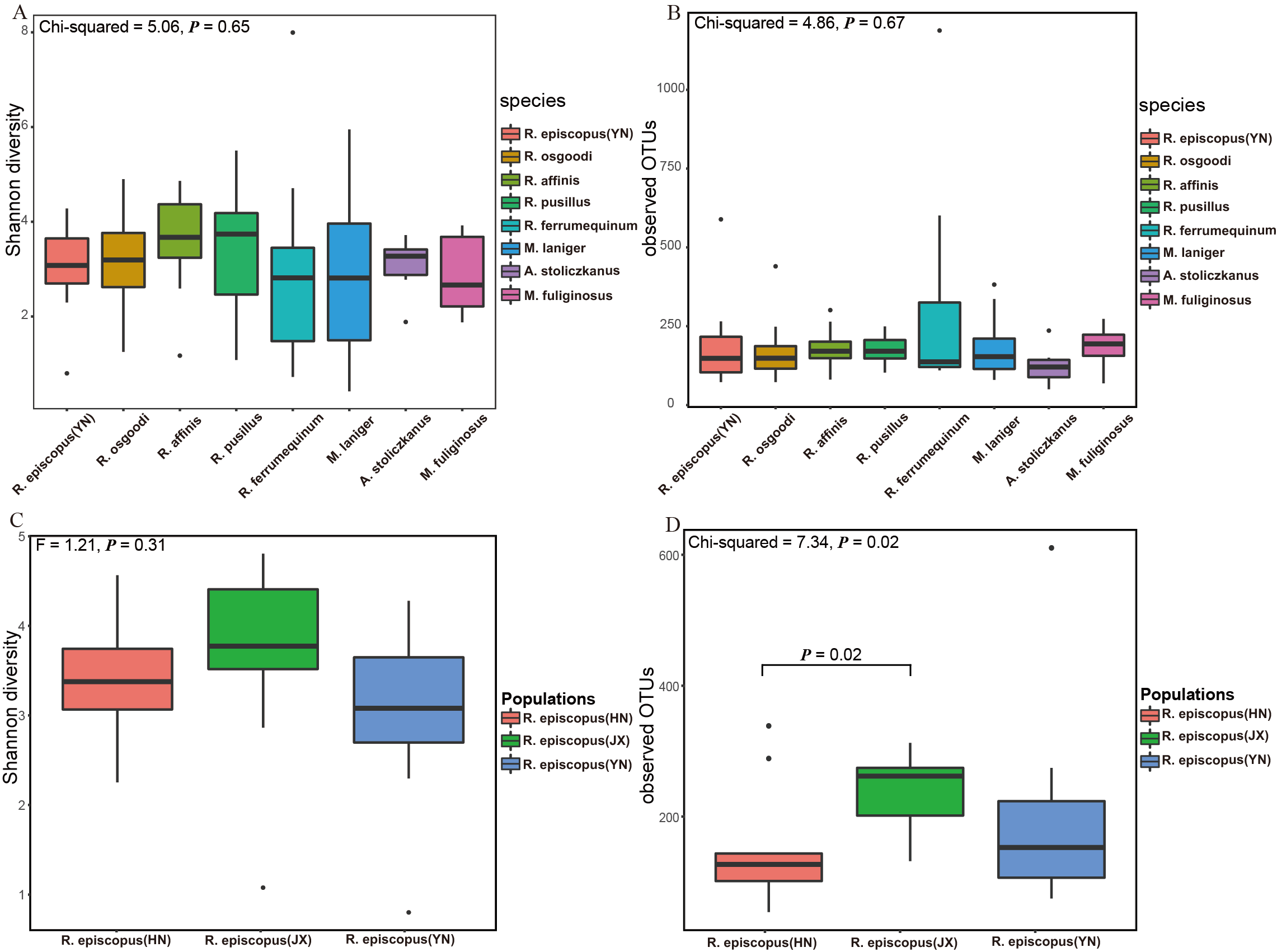

Supplement: FIG. S1 — Alpha diversity of gut microbiome among bat species. [file msphere.00087-24-s0001.tif]

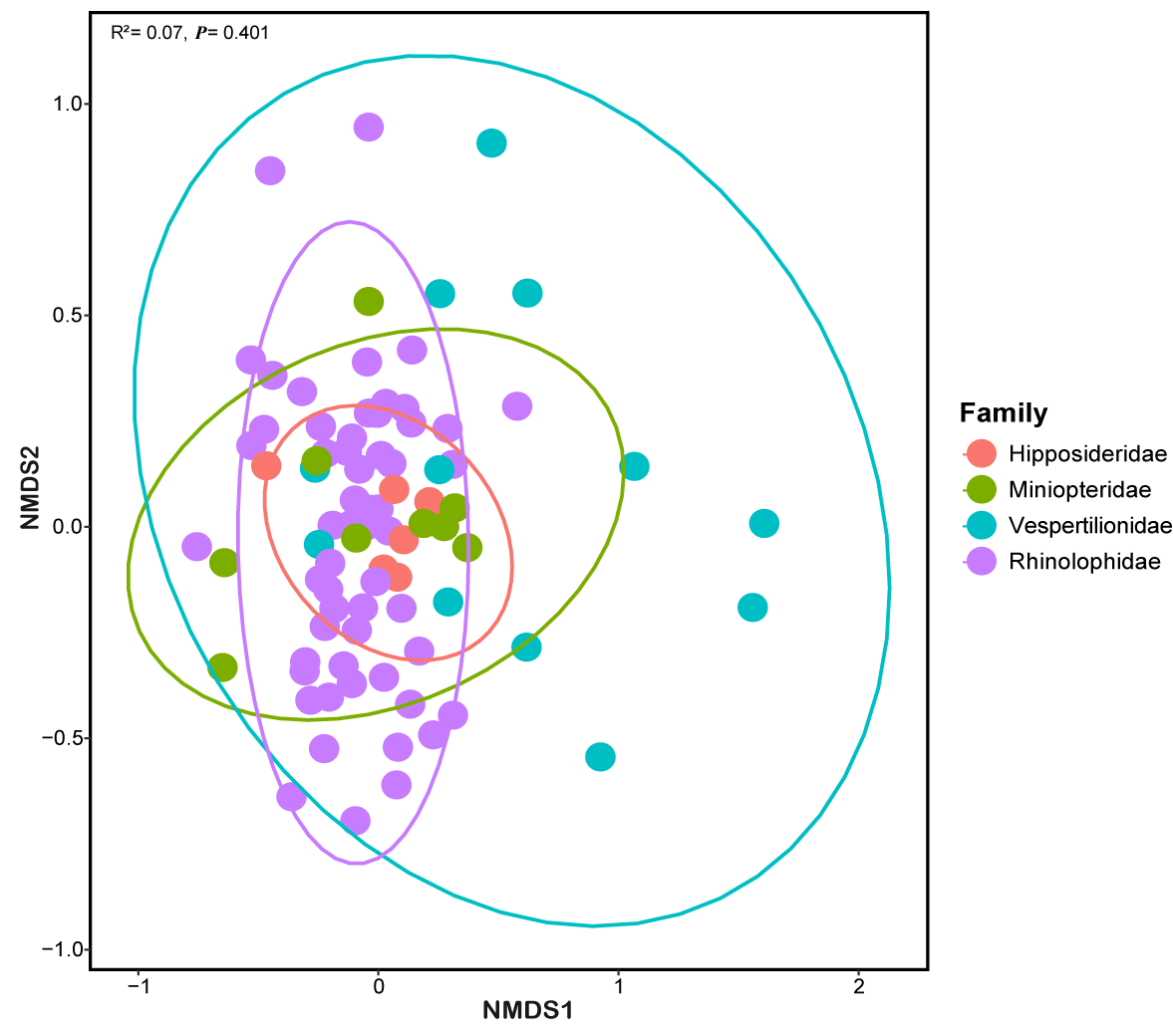

Supplement: FIG. S2 — NMDS results. [file msphere.00087-24-s0002.tif]

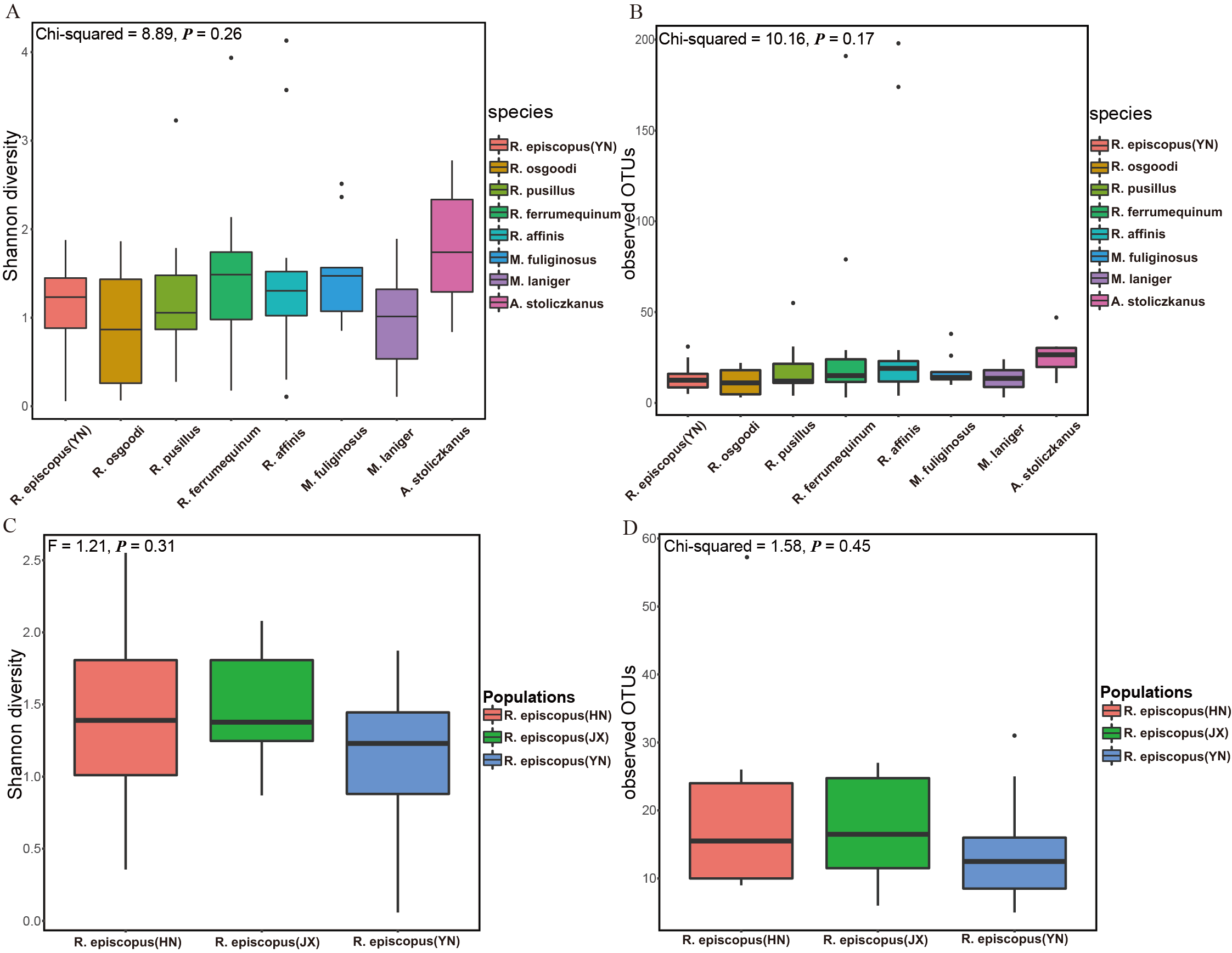

Supplement: FIG. S3 — Alpha diversity of diet composition among bat species. [file msphere.00087-24-s0003.tif]
