# Supplementary material for: The role of host traits and geography in shaping the gut microbiome of insectivorous bats
Source: mSphere. 2024 Mar 21;9(4):e00087-24. doi: 10.1128/msphere.00087-24 (PMC11036801; doi:10.1128/msphere.00087-24)
Supplement: Supplemental material legends — Legends for all supplemental items. [file msphere.00087-24-s0004.docx]

Supplemental material legends of all supplemental items

**FIG S1** Alpha diversity of gut microbiome among bat species. (A) Shannon diversity between all species, (B) Observed OTUs between all species, (C) Shannon diversity between *R. episcopus* populations, (D) Observed OTUs between *R. episcopus* populations.

**FIG S2** Nonmetric multidimensional scaling analysis of the beta diversity of the gut microbiomes at family level.

**FIG S3** Alpha diversity of diet composition among bat species, (A) Shannon diversity between all species, (B) Observed OTUs between all species, (C) Shannon diversity between *R. episcopus* populations, (D) Observed OTUs between *R. episcopus* populations.

**TABLE S1** Genbank accession numbers of the cyt *b* gene used in this study

**TABLE S2** Results of Kruskal–Wallis tset and analysis of variance (ANOVA) examining the differences in the gut microbiome at the phylum and genus levels among all bat species and *R. episcopus* populations

**TABLE S3** Pairwise PERMANOVA results of the gut microbiome at the species and family levels

**TABLE S4** Results of Kruskal–Wallis test and analysis of variance (ANOVA) examining the differences in diet composition at the phylum, family, and genus levels among all bat species and *R. episcopus* populations

**TABLE S5** Effects of predictor variables on gut microbiome of bats based on the best-fitting generalized linear model

**TABLE S6** Results of the Mantel test between the bat species with geographic overlap or similar diets
