# Supplementary material for: The role of host traits and geography in shaping the gut microbiome of insectivorous bats
Source: mSphere. 2024 Mar 21;9(4):e00087-24. doi: 10.1128/msphere.00087-24 (PMC11036801; doi:10.1128/msphere.00087-24)
Supplement: Table S1 — GenBank accession numbers. [file msphere.00087-24-s0005.docx]

Table S1 Genbank accession numbers of the cyt *b* gene used in this study

| Species | Country | Genbank accession numbers |
| --- | --- | --- |
| *Rhinolophus episcopus* | China | MN077591 |
|  | China | MN077637 |
|  | China | MN077571 |
|  | China | MN077576 |
| *Miniopterus fuliginosus* | Japan | AB085735 |
|  | China | MG570073 |
|  | Germany | MW684367 |
|  | Germany | MW684371 |
| *Aselliscus stoliczkanus* | China | DQ888670 |
|  | China | DQ888676 |
|  | China | EU434953 |
|  | China | EU434954 |
| *Myotis laniger* | China | EF555229 |
|  | China | EF555232 |
|  | China | MF630873 |
|  | China | OP245425 |
| *Rhinolophus osgoodi* | China | MN077624 |
|  | China | MN077623 |
|  | China | MN077643 |
|  | China | MN077640 |
| *Rhinolophus ferrumequinum* | China | DQ351848 |
|  | China | KX237527 |
|  | China | EU434936 |
|  | China | KR346932 |
| *Rhinolophus affinis* | China | DQ297582 |
|  | China | DQ987605 |
|  | China | KX467577 |
|  | China | MG570063 |
| *Rhinolophus pusillus* | China | EF217392 |
|  | China | MF630878 |
|  | China | DQ297577 |
|  | China | DQ297595 |
