# Supplementary material for: The role of host traits and geography in shaping the gut microbiome of insectivorous bats
Source: mSphere. 2024 Mar 21;9(4):e00087-24. doi: 10.1128/msphere.00087-24 (PMC11036801; doi:10.1128/msphere.00087-24)
Supplement: Table S5 — GLM result. [file msphere.00087-24-s0009.docx]

Table S5 Effects of predictor variables on gut microbiome of bats based on the best-fitting generalized linear model

| Dependent variable | AICc | predictors | estimate | z | *p* |
| --- | --- | --- | --- | --- | --- |
| Gut microbiome | 6475.17 | elevation | -0.0001 | -9.07 | < 0.0001 |
|  |  | Blattodea | 1.27 | 12.97 | < 0.0001 |
|  |  | Coleoptera | -0.19 | -3.62 | 0.0002 |
|  |  | Diptera | -0.20 | -8.21 | < 0.0001 |
|  |  | Miniopteridae | 0.23 | 4.98 | < 0.0001 |
|  |  | Rhinolophidae | 0.45 | 11.61 | < 0.0001 |
|  |  | Vespertilionidae | 0.61 | 12.81 | < 0.0001 |
|  |  | Hemiptera | -0.81 | -8.87 | < 0.0001 |
|  |  | Hymenoptera | -0.80 | -5.22 | < 0.0001 |
|  |  | latitude | -0.22 | -16.38 | < 0.0001 |
|  |  | Neuroptera | 0.59 | 4.62 | < 0.0001 |
|  |  | weight | 0.02 | 17.34 | < 0.0001 |
| Gut microbiome | 4441.05 | elevation | -0.0002 | -8.42 | < 0.0001 |
|  |  | Chironomidae | -0.67 | -8.02 | < 0.0001 |
|  |  | Cossidae | 1.64 | 36.82 | < 0.0001 |
|  |  | Crambidae | -0.12 | -2.16 | 0.03 |
|  |  | Culicidae | -0.97 | -13.23 | < 0.0001 |
|  |  | Dytiscidae | -0.63 | -8.48 | < 0.0001 |
|  |  | Erebidae | -0.31 | -6.93 | < 0.0001 |
|  |  | Miniopteridae | 0.34 | 7.37 | < 0.0001 |
|  |  | Rhinolophidae | 0.47 | 11.44 | < 0.0001 |
|  |  | Vespertilionidae | 0.52 | 10.26 | < 0.0001 |
|  |  | Geometridae | -0.52 | -9.95 | < 0.0001 |
|  |  | Lasiocampidae | -0.17 | -4.11 | < 0.0001 |
|  |  | latitude | -0.18 | -13.65 | < 0.0001 |
|  |  | Limoniidae | -0.23 | -5.60 | < 0.0001 |
|  |  | Muscidae | -0.66 | -15.06 | < 0.0001 |
|  |  | Noctuidae | -0.43 | -11.28 | < 0.0001 |
|  |  | Nymphalidae | -0.84 | -9.43 | < 0.0001 |
|  |  | Psychodidae | -0.23 | -4.42 | < 0.0001 |
|  |  | Saturniidae | -0.37 | -5.11 | < 0.0001 |
|  |  | Tipulidae | -1.24 | -8.44 | < 0.0001 |
|  |  | weight | 0.005 | 2.30 | 0.021 |
| Gut microbiome | 4835.46 | elevation | -0.0002 | -10.25 | < 0.0001 |
|  |  | Capua | -0.45 | -6.58 | < 0.0001 |
|  |  | Condica | -1.24 | -10.78 | < 0.0001 |
|  |  | Culex | -0.51 | -8.46 | < 0.0001 |
|  |  | Dinumma | -0.80 | -5.11 | < 0.0001 |
|  |  | Euproctis | 1.15 | 26.28 | < 0.0001 |
|  |  | Miniopteridae | 0.16 | 3.67 | 0.0002 |
|  |  | Rhinolophidae | 0.33 | 8.65 | < 0.0001 |
|  |  | Vespertilionidae | 0.35 | 7.38 | < 0.0001 |
|  |  | Laccophilus | -0.28 | -4.19 | < 0.0001 |
|  |  | Lacinipolia | -0.54 | -5.58 | < 0.0001 |
|  |  | latitude | -0.16 | -12.08 | < 0.0001 |
|  |  | Limonia | 0.26 | 5.85 | < 0.0001 |
|  |  | Peridroma | -0.18 | -3.29 | 0.0009 |
|  |  | Psychoda | 0.11 | 2.61 | 0.008 |
|  |  | Saturnia | 1.11 | 32.28 | < 0.0001 |
|  |  | weight | 0.006 | 3.67 | 0.0002 |

AICc: Akaike’s information criterion corrected for small sample size.
