# Supplementary material for: The role of host traits and geography in shaping the gut microbiome of insectivorous bats
Source: mSphere. 2024 Mar 21;9(4):e00087-24. doi: 10.1128/msphere.00087-24 (PMC11036801; doi:10.1128/msphere.00087-24)
Supplement: Table S6 — Mantel test. [file msphere.00087-24-s0010.docx]

Table S6 Results of the Mantel test between the bat species with geographic overlap or similar diets

| Comparison | r | *P* |
| --- | --- | --- |
| *A. stoliczkanus* - *M. fuliginosus* | 0.24 | 0.01 |
| *R. affinis* - *M. fuliginosus* | 0.15 | 0.01 |
| *R. ferrumequinum* - *M. fuliginosus* | 0.11 | 0.01 |
| *R. ferrumequinum* - *M. laniger* | 0.02 | 0.01 |
| *R. osgoodi* - *R. ferrumequinum* | 0.13 | 0.01 |
| *R. osgoodi* - *R. pusillus* | 0.35 | 0.01 |
| *R. pusillus* - *M. laniger* | 0.21 | 0.01 |
| *R. pusillus* - *R. affinis* | 0.25 | 0.01 |
| *R. pusillus* - *R. ferrumequinum* | 0.08 | 0.01 |
